# Foot pressure distributions during walking in African elephants (*Loxodonta africana*)

**DOI:** 10.1098/rsos.160203

**Published:** 2016-10-05

**Authors:** Olga Panagiotopoulou, Todd C. Pataky, Madeleine Day, Michael C. Hensman, Sean Hensman, John R. Hutchinson, Christofer J. Clemente

**Affiliations:** 1 Moving Morphology and Functional Mechanics Laboratory, School of Biomedical Sciences, The University of Queensland, Brisbane, Australia; 2 Institute for Fiber Engineering, Department of Bioengineering, Shinshu University, Ueda, Japan; 3 Adventures with Elephants, PO Box 1500, Bela Bela 0480, Limpopo, South Africa; 4 Structure and Motion Laboratory, Department of Comparative Biomedical Sciences, The Royal Veterinary College, Hatfield, UK; 5 School of Science and Engineering, The University of Sunshine Coast, Australia

**Keywords:** biomechanics, welfare, Proboscidea, locomotion, centre of pressure, elephant feet

## Abstract

Elephants, the largest living land mammals, have evolved a specialized foot morphology to help reduce locomotor pressures while supporting their large body mass. Peak pressures that could cause tissue damage are mitigated passively by the anatomy of elephants' feet, yet this mechanism does not seem to work well for some captive animals. This study tests how foot pressures vary among African and Asian elephants from habitats where natural substrates predominate but where foot care protocols differ. Variations in pressure patterns might be related to differences in husbandry, including but not limited to trimming and the substrates that elephants typically stand and move on. Both species' samples exhibited the highest concentration of peak pressures on the lateral digits of their feet (which tend to develop more disease in elephants) and lower pressures around the heel. The trajectories of the foot's centre of pressure were also similar, confirming that when walking at similar speeds, both species load their feet laterally at impact and then shift their weight medially throughout the step until toe-off. Overall, we found evidence of variations in foot pressure patterns that might be attributable to husbandry and other causes, deserving further examination using broader, more comparable samples.

## 1. Introduction

Elephant feet have evolved to resist locomotor stresses while carrying a large body mass (up to approx. 8000 kg in African elephants). Considering the importance of foot morphology for helping to support the body weight of elephants, the largest living terrestrial mammals, an understanding of any links between foot mechanics and the development of pathologies is vital. There have been important basic scientific studies that describe quasi-normal foot form and function in elephants. African elephants (like Asian species) walk on their ‘tip-toes’ (subunguligrade), yet their feet are effectively flat (i.e. functionally plantigrade) due to a large, compliant fat pad that fills the space behind the toes [1]. Elephants also have enlarged false toes called ‘predigits’ embedded in those fat pads that help transmit loads from the sole of the foot proximally up the limbs [2]. When compressed, the fat pad acts to evenly distribute locomotor stresses across the sole of the foot, especially behind the toes [3–5]. Evolutionarily, there appears to be a strong correlation between body mass, foot posture and development of a fat pad in elephants. The fossil record reveals that the foot bones in small-bodied early elephants (stem Proboscidea) were held relatively flat (near-plantigrade) and had no evidence of an enlarged fat pad; however, as elephants evolved larger body mass and became more terrestrial, their foot posture became more plantar-flexed and they evolved fat pads along with predigits [2]. Similar trends showing evidence for correlated evolution of larger foot pads, more digitigrade foot postures and increases of body mass have been proposed for ornithopod dinosaurs [6]. The presence of the fat pad in large-bodied land animals such as elephants may thus be a mechanism to reduce locomotor stresses that could impact the animals' health and welfare.

Panagiotopoulou *et al*.'s [4] study of *in vivo* foot pressures in captive Asian elephants showed that peak pressures (i.e. the maximum pressure over time at a particular location) were focused towards the tips of the outermost (lateral) toes (digits III, IV and V) where pathologies are most prevalent in elephants in general, and that the lowest pressures were in the middle and towards the rear of the feet, directly under the fat pad. Pressure reduction underneath the fat pad can be attributed to the compliance of the tissue but also to the observation that elephants minimize heel-only contact while walking, using mainly the front part of their feet to produce vertical forces as they roll over the fat pad during ground contact [4].

Foot anatomy and locomotor behaviour in elephants should reduce peak pressures that could cause foot disease. However, a major problem that hinders conservation efforts with elephants in captivity is that some elephants develop foot disorders, apparently uncommon in wild populations [7–10]. The causes of foot disorders in captive elephants are complex, with obesity, space availability and time spent on hard substrates being major suspects [11–14]. The latter suspicion is consistent with studies conducted on cattle that reported a higher incidence of lameness and pathogenesis in animals housed on hard surfaces (e.g. [15,16]). An investigation into the welfare and quality of elephants in zoos in the United Kingdom found a high prevalence of gait and foot abnormalities in animals kept in enclosures with unnatural, hard substrates [13]. In that study, 80.4% of the elephants included showed foot problems, ranging from cracks to infection, and 85.7% had abnormal gait, ranging from slightly abnormal to severely lame [13]. A recent study in Asian and African elephants in North American zoos using medical examinations, clinical records and demographic characteristics showed a close relationship between foot pathologies and the time the animals spent on hard substrates [14]. In particular, Miller *et al*. estimated that elephants exposed to hard substrates for 4 h daily were more likely to develop joint stiffness or limb lameness than those exposed to hard substrates for 2.5 h per day. Furthermore, within a population of 215 elephants that received physical examinations, 145 had foot abnormalities, with 92.4% showing nail abnormalities (such as inflammation, cracks, defects and horn growth), 13.1% with pad abnormalities and 22.8% with abnormalities in their interdigital spaces [14].

Management of foot disease in elephants is very challenging because treatment is often unsuccessful or it can have life-threatening effects. Physical examination using observations on gait abnormalities can be informative but solely when foot conditions have advanced [14]. Examination using diagnostic techniques is costly and can negatively affect animal health because it may require general anaesthesia (e.g. [17]) or other ‘hands-on’ methods that are impractical especially in settings (e.g. protected/non-contact) where interaction of captive elephants and their human keepers is minimal or absent. These challenges, coupled with the tendency for foot disease in elephants to become evident when it has progressed to irreversible levels, make euthanasia a frequent end result of foot disease [7–10,17]. Wildlife reserves and safari parks are also classified as captive settings; however, these tend to better mimic the natural habitats of elephants, often presumed to provide a healthier living environment. Elephants kept in larger outdoor spaces with natural terrain are thought to remain more athletic and have better health and gait compared with those in less spacious, more unnatural environments [13,14]. Elephants in natural environments exercise their feet by digging the ground, walking on rocks, and rubbing their pads against mud and sand pits (electronic supplementary material, movie S1) [18,19]. These behaviours help ensure that elephants' feet remain moist, their fat pads stay supple and their nails receive natural trimming [18,19]. Walking on unnatural substrates may dry out the sole (slipper) or encourage cracks to develop [14,18,19].

To prevent the development of foot diseases in captive elephants, especially in habitats with less natural substrates, many elephant keepers perform regular trimming on the feet [18,19]. Trimming is usually performed every three to four weeks, using sharp blades attached to handles. The amount of tissue removed during trimming depends on the thickening of the skin, according to the keepers' observations [11]. Trimming techniques are beneficial in cases of fungal growth, cracks and divots when performed locally; however, excessive trimming of the sole may have adverse effects such as inducing soft tissue infections (e.g. [20]). In addition, trimming approaches may vary between African and Asian species to take into consideration those elephants' adaptations to different environments (e.g. simply put, more savannah-based versus more forest-based) [19].

Although epidemiological and pathological studies have increased our understanding of the prevalence of foot diseases in captive elephants, they are based on observations and measurements that come from elephants that already have foot problems and/or from cadaveric tissues and museum specimens. In both cases, such data may come with limited background information and little or no direct mechanistic basis. It is still not known how changes in habitats' substrate properties or foot management influence locomotor pressures and foot pathogenesis in living elephants, or the biomechanical mechanisms underlying their formation, especially longitudinally across the lifetime of an elephant (approx. 80 years).

This study's primary aim is to quantify variations of the pressure patterns among the feet of African elephants, for comparison to existing data on Asian elephants [4]. Prior studies of these species showed that their locomotor mechanics are not appreciably different (e.g. [21,22]), and differences in their foot anatomy are minor [1,23]. For this reason, we do not expect to find drastic differences in the centre of pressure (COP) trajectories (i.e. mean paths of the vertical ground reaction forces on the feet) and pressure patterns between species. Thus, we predict that any variations in pressure patterns may be related to differences in their husbandry, including but not limited to trimming and the substrates they typically stand and move on.

## 2. Material and methods

### 2.1. Subjects

Five healthy adult African elephants from a safari park (Adventures with Elephants, Limpopo, Bela Bela, South Africa) were selected to participate in this study (table 1). The elephants' weights could not be measured during data collection and all body masses were thus based on approximations by the park keepers. The African elephants were kept under near-wild conditions in a 380 hectare grassland habitat with natural substrate properties and ample space to move and forage (electronic supplementary material, movie S1). As a result, all elephants seemed to naturally trim their feet and did not directly receive foot care from their keepers. The health of the animals' feet was visually assessed daily by their keepers during the safari interactions with tourists. It was not possible to collect data from wild African elephants due to experimental constraints and accessibility issues.

**Table 1.** Subject characteristics (*Loxodonta africana*) and number of experimental trials and steps. Circumferences for the right fore and hind feet (RFF, RHF) were measured twice by independent individuals and the mean is shown here. Mean ± s.e. given for each mean peak (maximum) pressure during the whole stance.

	subject 1	subject 2	subject 3	subject 4	subject 5
age (years)	15	18	11	12	13
sex	M	M	F	F	F
body mass (kg)-estimated	2800	3000	1500	1800	2300
shoulder height (m)	2.43	2.50	2.22	2.12	2.30
RFF circumference (m)	1.21	1.27	1.06	1.06	1.08
RHF circumference (m)	1.18	1.24	1.07	1.10	1.08
mean Froude number	0.07	0.05	0.07	0.08	0.08
mean velocity (ms^−1^)	1.24	1.11	1.24	1.24	1.34
mean maximum pressure (kPa) fore left	317 ± 68	251 ± 23	256 ± 10	262 ± 16	315 ± 19
mean maximum pressure (kPa) fore right	296 ± 24	312 ± 29	236 ± 10	224 ± 10	318 ± 21
mean maximum pressure (kPa) hind left	237 ± 16	250 ± 10	243 ± 12	200 ± 11	297 ± 27
mean maximum pressure (kPa) hind right	244 ± 18	248 ± 12	251 ± 13	235 ± 14	284 ± 44
number of steps	28	29	76	48	29
number of trials	13	18	30	23	10

The Asian elephants in Panagiotopoulou *et al*. [4] were reared in more typical captive safari park environments from two zoological parks in Bedfordshire, UK (the Zoological Society of London's Whipsnade Zoo and Woburn Safari Park) and received regular foot trimming for disease prevention. Their age and body mass at the time of data collection varied from 31 days and 120 kg to 27 years and 3332 kg.

### 2.2. Data collection

**Figure 1.**
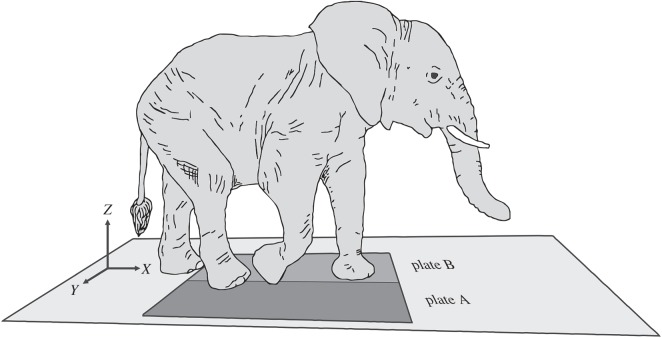
Schematic illustration of the position of the pressure plates during data collection.

Experiments were conducted at the safari park using two pressure platforms that were specifically built to dynamically measure pressure distributions and COP trajectories of elephants for this study (Zebris Medical GmbH, Biomechanix, Munich). Each platform had sampling frequency of 100 Hz, and outer dimensions 0.605 m width × 2.122 m length. Each platform contained an array of sensors arranged 44 × 160 to total 7040 sensors per platform (14 080 sensors in total; approx. 0.55 sensors cm^−2^). The platforms used in this study were nearly twice as large as the pressure platform used in previous studies on Asian elephants, allowing the collection of a greater number of steps, but had lower resolution than this previous study ([4]; 1.0 m × 0.4 m, 8192 sensors, 2.05 sensors cm^−2^). The pressure platforms were calibrated as per manufacturer's instructions and were aligned parallel to each other, with their longitudinal axes aligned to the direction of travel (figure 1). This allowed measurements of multiple footfalls from a single stride, maximizing the collection of spatially complete data, while accounting for the animals' wide gait. Both platforms were placed on levelled natural ground and covered with 0.5 mm thick rubber matting in order to avoid recognition of the plates' locations by the animals. Pressure readings were zeroed after being covered by the rubber matting and prior to starting each data collection session.

All elephants were trained to walk over the set-up prior to the experiments. Training lasted approximately 1 h, during which time each animal was guided by the keepers to walk over the set-up five times. Prior to data collection, reflective markers were placed on the elephant's body at shoulder and hip height for the recording of speed. Walking speed was recorded using an uncalibrated GoPro Hero 3 (GoPro Inc., San Mateo, California, USA) camera at 24 Hz sampling frequency. The camera was placed approximately 2 m away from the platforms and faced perpendicular to the walkway. We did not collect data from running gaits as they were deemed unsafe, and comparative foot pressure data do not exist for such gaits in Asian elephants. All unstable or unsteady walking trials, such as when the elephants stopped mid-way, accelerated/decelerated or walked in an obviously abnormal manner, were excluded from further analysis.

During the experiments, the elephants were guided by their keepers to walk over the walkway an average of 40 times each (electronic supplementary material, movie S2). The experimental approach was similar to that previously used by Panagiotopoulou *et al*. [4] and did not cause any discomfort to the animals.

### 2.3. Data processing

Initial data analysis was conducted in Canopy v. 1.4.1 using SciPy v. 0.14, NumPy 1.8.1 and Matplotlib 1.4 (Enthought Inc., Austin, TX, USA). The raw pressure data (*x*, *y*, time) of all individual footsteps were exported from the Zebris system, isolated algorithmically using spatio-temporal gaps between clusters of non-zero pressure voxels and were assessed for spatio-temporal completeness as per Panagiotopoulou *et al*. [4]. Spatio-temporally complete images were then identified as manus (forefeet) or pes (hindfeet) and right/left. For homologous comparisons between footsteps and within feet, all images were registered (aligned) manually using a computer keyboard [24]. Existing automatic pedobarographic registration algorithms that have been previously developed for human feet [24] do not perform well on elephant data due to their symmetrical shapes. Manual registration has been shown to provide results that are as good as optimal algorithmic registration [24], and we previously used this method in a foot pressure study on captive Asian elephants [4]. The template image (isocontour of threshold 5 kPa), with which all other images were aligned, was the first image to meet the inclusion criteria (see Panagiotopoulou *et al*. [4]: Material and Methods). Source (non-template) images were manually translated and rotated using the keyboard to align them with the template of each foot (fore left (FL), fore right (FR), hind left (HL), hind right (HR)). The translation and rotation manually applied to each source image were re-applied to the pressure images' time series.

**Figure 2.**
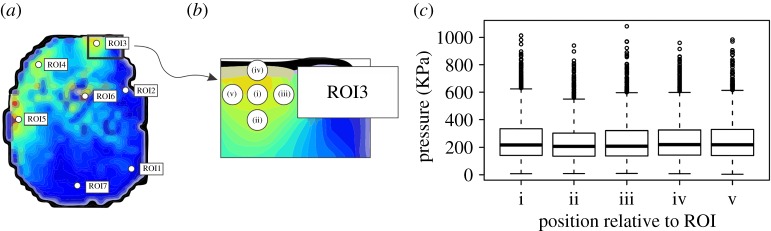
Sensitivity analysis for the placement of each region of interest (ROI) in a representative left fore foot (Subject 1). (*a*) Placement of the seven ROIs. (*b*) Detailed section of the area around ROI3, showing the placement of each sensitivity analysis point around it. (*c*) Box plot showing the effect of placement among all trials and subjects (*n* = 7350). Boxes represent the median, with hinges representing the first and third quartiles. Whiskers represent the 95% CIs, and dots represent outliers.

Following registration, seven anatomical regions of interest (ROIs) were selected on the mean images as per Panagiotopoulou *et al*. [4] (figure 2*a*). The ROIs represented chosen homologous anatomical structures of the African elephants' feet where peak pressures of the whole stance phase were extracted for analysis. ROIs 1–5 represented the middle of the bottom surfaces of the nails of digits i–v, respectively, ROI 6 represented the middle of the foot sole (slipper) and ROI 7 represented the caudal-most (heel) aspect of the sole. The peak pressures (kPa) per subject, trial and foot were extracted from a 3-pixel area for each digitized ROI using a Gaussian kernel with a standard deviation of one pixel.

Manual identification of ROIs is a standard approach to calculate pressure magnitudes in functional studies but can be biased because single values do not necessarily represent neighbouring data [24]. To account for this limitation, we conducted a sensitivity analysis to evaluate the effect of ROI location by moving the selected points by one pixel. To do this, we defined another four points, directly above, below, to the left and right of each ROI (figure 2*b*) and estimated the sensitivity to digitizing error by using a linear mixed effects model using the lmer.R function from the lmerTest package [25] in R v. 3.1.1 software (R Development Core Team, Auckland, New Zealand). The random effects of subject, foot and ROI were included. The *P* threshold was set at 0.05 and all significance values were estimated using the Satterthwaite's approximation, as implemented in lmerTest.

To measure peak foot-pressure magnitudes during the stance phase and to determine pressure variations among ROI and feet, we similarly used a linear mixed effect model including subject as a random effect in R. To compare variations between feet, we calculated the mean COP trajectories for each foot and performed a pair-wise comparison within feet using Pearson's correlation along the cranio-caudal axis (*Y r*) and the mediolateral axis (*X r*), the root mean square error (RMSE) on untransformed data, and the root mean square deviation on transformed data (RMSD). Data were transformed using a Procrustes analysis in R using the function procOPA.R from the shapes package [26], with both scale and reflect set to TRUE.

The COP data from the African elephants were then compared with the COP data from Asian elephants collected using similar methods from Panagiotopoulou *et al*. [4]. Means were calculated for each foot from each dataset, excluding the juvenile subject 1 (120 kg) from the Asian elephant dataset, considering its different COP trajectories when compared against the other Asian elephant subjects, probably due to its young age [4]. Other subjects were not excluded, although the Asian subjects 2 and 3 were younger than the African elephants in our new sample and thus smaller (500 and 1024 kg body masses), so age differences might contribute to other factors underlying any differences between elephants.

Considering the likely differences in feet circumferences between the two species due to size and trimming parameters, a direct comparison between the two species was not possible. For this reason, all data were transformed using a Procrustes analysis in R as above, which allowed the COP trajectory to be scaled and reflected to maximize similarity. Unlike the analysis among feet, which compared both the original and transformed data, this analysis used only the transformed values in the comparison between African and Asian elephants.

## 3. Results

The mean walking speed of all African elephants was 1.2 ms^−1^ (table 1). This corresponded to a Froude number ([27]; Fr = velocity^2^ × [9.81 ms^−2^ × hip height]^−1^) of 0.07 for all subjects (table 1), indicative of a slow walk (electronic supplementary material, Data S1). This speed was comparable with the mean speed of the Asian elephants in Panagiotopoulou *et al*. [4] (mean Fr = 0.10).

The ROI sensitivity analysis indicated a significant effect of the topological location of each ROI on peak pressure magnitudes (*F*_4,7314_ = 8.22, *p* < 0.001) (electronic supplementary material, Data S2). An ANOVA implemented using the aov.R function also indicated a significant interaction between the ROI placement location, with both foot (*p* < 0.001) and ROI (*p* < 0.001). A Tukey *post hoc* test showed that this effect was largely a result of position ii (ii in figure 2*b*) showing significantly lower pressures than each other position (figure 2*c*). Interactions between other positions (i, iii, iv, v) were not significant (*p* > 0.05). To account for the effect of the topological location of each ROI on pressure magnitudes, the mean peak pressure value for each topological location and each ROI was used for further processing.

To determine the variation among ROIs and among feet, we used a linear mixed effect model including subject as a random effect. Mean peak pressure during the whole stance phase varied significantly with both the ROI (*F*_6,1456_ = 403, *p* < 0.001) and the foot (*F*_3,1459_ = 6.58, *p* < 0.001), with a significant interaction between the terms (*F*_18,1438_ = 25.8, *p* < 0.001). Mean peak pressure among all feet was variable, with the front feet tending to support more pressure. When combined into fore and hind pairs, the forefeet showed significantly higher mean peak foot pressures based on ANOVA (fore 264 ± 5.31, hind 246 ± 5.09; *F*_1,1468_ = 5.22, *p* = 0.022). This finding, although only a mean 7% difference between the fore and hind foot pressures, is loosely in accord with the observation that elephants' forelimbs carry more weight; approximately 60% of the animal's body weight [28–31]. A *post hoc* test among all feet showed a significant difference between the FL versus HL and the FL versus HR, with no other interaction being significant.

**Figure 3.**
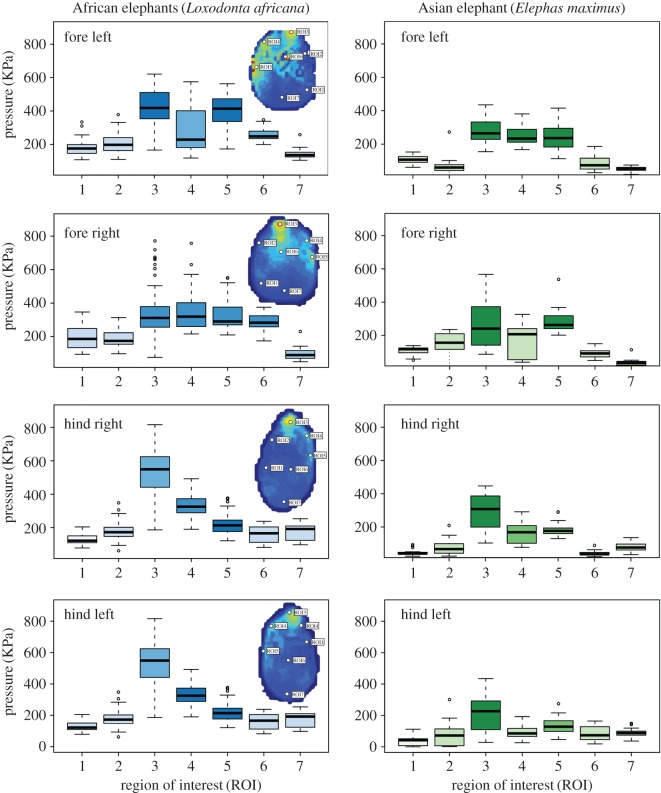
Pattern of mean peak pressure distributions during the whole stance phase for the feet of the African elephants (left; this study) and Asian elephants (right; [4]). Regions of interest (ROIs) are shown for each foot, and the variation among them is shown in the box plots. Where possible, we have attempted to show statistically similar ROIs through similar colour shadings. Boxes represent the median, with hinges representing the first and third quartiles. Whiskers represent the 95% CIs, and dots represent outliers.

**Figure 4.**
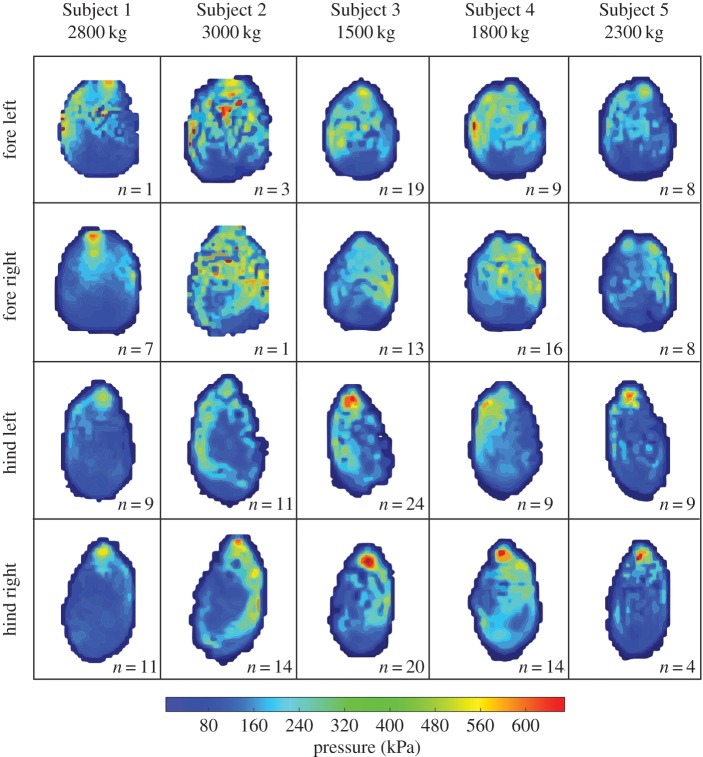
Means of the peak pressure patterns created from the peak pressure sample during the whole stance phase for each subject and each foot. Peak pressure patterns shown here were smoothed (using a Gaussian blur) to interpolate between pressure grid points.

Peak pressures were also compared across ROIs using ANOVA with *post hoc* tests. When combined in a single analysis, all ROIs displayed significant variation from one another with the exception of regions 1 versus 7 (*p* = 0.707), 2 versus 6 (*p* = 0.131) and 4 versus 5 (*p* = 0.171). However, as indicated by the significant interaction between ROI and foot, this pattern did show some variation among the feet (figure 3). ROIs 3–6 of the forefeet showed moderately higher mean peak pressures than regions 1, 2 and 7. The hind feet, however, presented the significantly highest mean peak pressures in digit III (ROI 3), with the lateral digits (ROI 4, 5) showing moderate pressures, and the medial digits (ROI 1, 2) and the central (ROI 6) and caudal (ROI 7) aspects of the sole exhibiting the lowest pressures (figures 3 and 4).

The mean peak pressures during the whole stance phase among all ROIs, feet and subjects in the African elephants were 946 kPa (electronic supplementary material, Data S2). The mean peak pressure value from the Asian elephants in Panagiotopoulou *et al*. [4] was 567 kPa after excluding Subject 1 (electronic supplementary material, Data S3), indicating that the mean peak pressures of our African elephants were almost 1.7 times those of the Asian elephants sampled.

**Figure 5.**
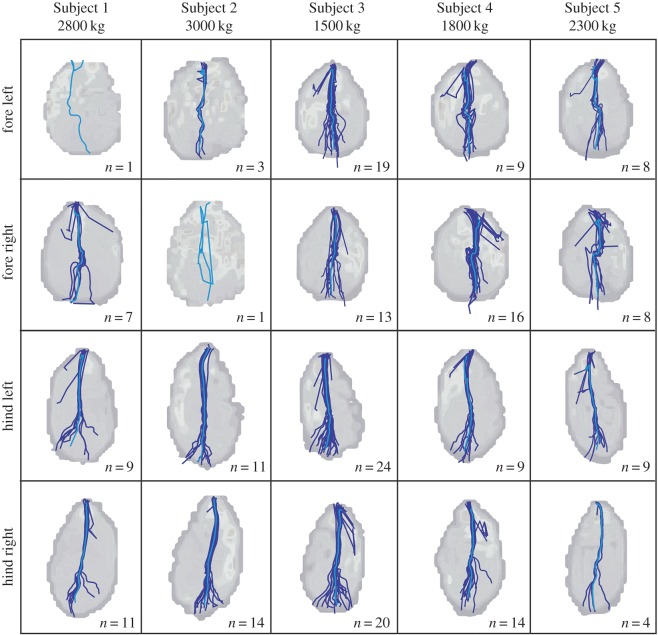
The patterns of the centre of pressure (COP) throughout the stance phase for each foot of each subject. Individual trials are indicated in dark blue, with the mean of all trials indicated with the lighter blue line.

The COP trajectories of the African elephant feet throughout the stance phase are shown in figure 5 (for raw COP data see electronic supplementary material, Data S4). COP traces were variable among subjects and among feet. The greatest variation was evident between the fore and hind feet. The patterns of the COP trajectories were the best conserved between the hind feet pairs, at least when COP shape was transformed via the Procrustes analysis using the function procOPA.R from the shapes package [26], with both scale and reflect set to TRUE (table 2). Among the untransformed data, the forefeet showed the lowest variation. Almost all of the variation between the pairs was around the medial-lateral axis, with a more predictable heel-to-toe, caudal-to-cranial movement of the COP present in all feet.

**Table 2.** Pair-wise comparisons between different feet for the African elephant subjects. The comparison included Pearson's correlations along the cranial-caudal axis (*Y r*) and the medial-lateral axis (*X r*), the root mean square error (RMSE) for untransformed data, and the root mean square deviation for transformed data (RMSD).

pair-wise comparison	*X r*	*Y r*	RMSE	procOPA.R RMSD
FL–FR	−0.76	0.97	1.613	1.38
FL–HL	−0.53	0.98	1.797	1.15
FL–HR	−0.14	0.98	2.361	1.26
FR–HL	0.21	0.91	2.934	1.91
FR–HR	0.21	0.91	2.621	1.95
HL–HR	−0.59	0.99	2.219	0.39

**Figure 6.**
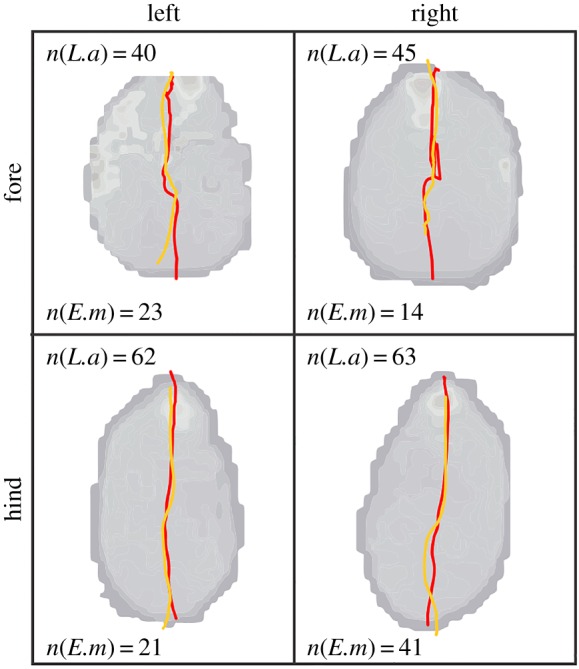
Comparison between the mean COP patterns of the African elephants (red) and the Asian elephants (orange; cf. [4, fig. 9]). Data for the Asian elephants were transformed via Procrustes analysis in R using the function procOPA.R from the shapes package [25], with both scale and reflect set to TRUE. *L.a* and *E.m* respectively refer to *Loxodonta africana* and *Elephas Maximus*.

The COP comparisons between the African elephants from this study and the Asian elephants from Panagiotopoulou *et al*. [4] showed similar trajectories (figure 6). In both species, the caudal-to-cranial pattern of movement predominated, with small lateral deviations at foot impact (table 3).

**Table 3.** Pair-wise comparisons between African and Asian elephants using transformed values of COP paths. Values were transformed via Procrustes analysis in R.

pair-wise comparison	*X r*^T^	*Y r*^T^	RMSE^T^	RMSD
FL	0.17	0.99	0.548	0.77
FR	0.52	0.96	0.852	1.21
HL	0.42	0.99	0.752	1.06
HR	0.84	0.99	0.858	1.21

## 4. Discussion

This study characterized locomotor pressure distributions over discrete foot regions (ROIs) in walking African elephants reared in near-wild captive conditions in Africa and compared these data with previously published data on Asian elephants [4] reared in more typical captive (zoo/park) environments in the United Kingdom. Our records of mean peak pressures corresponded to a fairly typical (if slightly slow) walk of 1.2 ms^−1^. Pressures are expected to increase at faster speeds, although our limited samples of Asian elephants [4] at Fr ∼ 0.1 and African elephants (this study) at Fr ∼ 0.07 instead showed higher pressures in (slower-moving) African elephants, so across this narrow speed range at least, pressure may not change greatly. While the uncalibrated GoPro camera may have influenced the speed, Hutchinson *et al*. [21] found that elephants often self-selected similar speeds (Fr ∼ 0.1), which may seem slow but larger animals tend to choose relatively slower speeds as per [32]. We found that during slow walking both species showed similar COP trajectories. We also recorded the highest mean peak pressure values during the whole stance phase for the African species on the third, fourth and fifth digits of both the fore and hind feet, yet pressures on the forefeet were higher, related to bearing more weight (figure 3). This finding is similar to the Asian elephants' results from Panagiotopoulou *et al*. [4] (figure 3).

While the ROI approach is a useful technique to measure pressures on specific foot regions, it overlooks intra-region variability by assuming that all zones are functionally independent. Our sensitivity analysis showed a significant interaction between the topological location of each ROI and the pressure magnitudes (figure 2), confirming that the ROI approach underestimates intra-region variability and can influence pressure magnitudes, making the inclusion of sensitivity analysis to measure the degree of variation an essential prerequisite. Nevertheless, this limitation did not influence the credibility of this study, which is focused on the overall similarities and differences in pressure patterns between the two samples of elephants, rather than the exact pressure magnitudes.

Direct comparisons of pressure magnitudes between the Asian and African elephant species showed an approximately 1.7× increase in mean peak pressures in the African species, compared with the Asian participants from Panagiotopoulou *et al*. [4]. However, it is likely that this difference is at least partly caused by disparities in the body masses of the animal participants and the equipment differences between the two experiments. Some of the Asian and African participants had similar body masses, yet the African sample had a higher number of spatially and temporarily complete steps due to the larger width and length of the pressure plates. Taking sample numbers from each individual into account results in a weighted average mass of 2060 kg for African elephants, but only 1156 kg for the Asian elephants, owing to the largest individuals of the latter species being represented by the fewest trials [4]. This difference probably explains the variation in the absolute pressure magnitudes between the studies. Given that body mass values for African elephants here were estimates only, we were reluctant to provide body mass-corrected values for comparison. The differences in the foot management of the two species may also have influenced pressure distributions, yet more rigorous comparisons of captive substrates and foot-trimming protocols using controlled experiments are needed for statistically sound conclusions to be drawn and strongly evidence-based animal husbandry guidelines to be established.

The pressure pattern comparisons between the two species showed that both African and Asian elephant samples endured the greatest pressures on the forefeet. In addition, the mean peak pressures for both samples were concentrated on the lateral toes (digits iii, iv, v), where foot diseases are usually found, and the minimum pressures were on the medial toes and under the digital cushion [4, figs 5 and 8]. This finding supports our hypothesis that the two species' samples will have similar foot pressure patterns mainly due to their similarities in foot anatomy [23] and locomotor mechanics [21,22]. Anatomically, differences in the feet of the two species are mainly restricted to the number of toenails [20]. While African elephants tend to have four toenails on their forefeet, Asian elephants have five toenails on the manus and four on the pes [19]. Biomechanically, the differences between general limb function in African and Asian elephants appear to be negligible [21,33].

The similarities in locomotion between the two species are further supported by the similarities in the COP trajectories we measured in this study (figure 6). Overall, both species loaded their feet more laterally at impact, shifted their weight medially at mid-stance and the COP followed the mid-central sole during toe-off. Thus, the lack of peak pressures on the caudal part of the foot (around the heel) can be attributed to the fact that elephants minimized heel contact while walking, concentrating pressures on the front part of their feet. It has been hypothesized that the pressures on the central part of the foot are minimal due to the presence of the compliant fat pad that fills the space behind the toes to evenly distribute locomotor stresses across the central part of the sole, and potentially acts as a venous pump [1,4,34].

Elephants often need to be kept in captive enclosures for protection from poachers or other threats, as well as for touristic and educational purposes. Our current and previous [4] studies have shown that elephants kept in captive environments with natural substrates that may imitate the diversity of their natural habitats concentrate mean peak pressures on the lateral digits (regardless of species) and maintain the lowest pressures around the fat pad. Captivity in enclosures with hard ground such as concrete or tarmac may prevent animals from moving their distal limb joints and stretching various connective tissues the same ways they can in more wild conditions (e.g. [19]). As a result, the locomotor pressures on their lateral digits may increase and thus pathogenesis of foot diseases might, in some circumstances, be accelerated (e.g. [14,17]). Despite some differences in management regimes and other aspects of the elephants in the two samples that we have focused on in this study, we found some remarkable similarities in how these elephants load their feet. However, we also found more subtle (or ambiguous) differences whose causes are too uncertain to draw conclusions from.

Our sample size (as in [4]) was admittedly small; nevertheless, despite the small sample size there was sufficient statistical power to infer differences among the ROIs. Null results including ANOVA interactions could have been secondary to small sample size and inadequate statistical power, but because our hypotheses did not pertain directly to those effects we believe that sample size and power were adequate for this study. There are no currently published data that indicate the optimal sample size to use in locomotor studies on foot pressures. Sample size depends largely on a number of factors including variability of the traits measured and accessibility of the animal participants. In this study, we have measured the maximum number of elephants available. Comparisons of small numbers of individuals are of course limited statistically, constituting more of a descriptive/anecdotal sample than a truly robust one in statistical terms, but we have reported the variability in our data, and are conservative with our comparisons and conclusions. We seek to describe broad patterns of movement in these species, and describe interesting similarities and differences. Larger sample sizes of more elephants with controlled environments and related parameters, maximizing comparability of those samples, are needed to fully test how different management regimes, habitats, ages, body weights, species' evolutionary backgrounds or other factors influence the normal and pathological functioning of elephant feet, which could lead to future improvements in elephant welfare.

Despite its limitations, this study is the first to show that, regardless of species, captive elephants reared in outdoor spaces with natural substrates and sufficient space for the animals to remain athletic, load the most the lateral aspect of their feet while walking. This loading pattern coupled with the reduced pressure below the fat pad seems to be an effective mechanism for large-bodied mammals to maintain foot functional integrity and potentially balance while carrying their large body mass. Nevertheless, while this mechanism works well for animals reared in wild and semi-natural habitats, it may not be effective in captive conditions where unnatural substrates predominate [11–14]. To date, it is not clear how hard substrates can disrupt the mechanics of elephant feet but elephants kept in confined spaces for long hours or housed in small yards with hard substrates (asphalt and concrete) are more prone to foot disease than their counterparts reared in semi-wild environments [14,18,19]. Confined environments with surfaces covered in lingering faeces and urine can exacerbate infections around the pads and nails [18]. We speculate that captivity-induced foot infections around the fat pad will influence the compliance of the fat pad tissue and reduce its ability to dissipate locomotor pressures. An infected or cracked fat pad will thus potentially increase loading on the distal part of the lateral digits of the elephant's foot and accelerate foot disease.

Foot disease in elephants is not always detectable at its early stages using physical examinations of the foot. However, the techniques used and the hardware developed in our study (also [4]) could provide a new diagnostic measurement that can be integrated by veterinarians or keepers to monitor elephant foot health, in the same way that podiatrists use pressure mapping to diagnose and treat human foot disorders. Elephants, which trim their feet naturally by walking in diverse substrates, are expected to load more the lateral aspect of their foot at impact and keep pressures low below the fat pad. Changes in the COP trajectories, patterns and magnitudes within individuals may be indicative of pain or early stages of foot disease. The use of our proposed methodology to regularly monitor foot pressure trajectories and magnitudes in captive elephants might allow animal keepers to detect abnormalities at early stages (when foot problems are not evident with physical examination) and create subject-specific foot management protocols that can prevent or slow down disease progression. Such protocols could include different trimming techniques for captive elephants. Trimming is a popular foot care approach to remove any cracks and divots in cases when elephants walk in less natural habitats. Nevertheless, trimming protocols vary and their effect on the foot function is still unknown. This is particularly uncertain when trimming is conducted on the whole sole rather than on concentrated areas. Better monitoring of the foot pressures in captive elephants using pressure platforms may allow keepers to decide upon subject-specific trimming protocols that will remove cracks and overgrowths but will not affect foot mechanics. In addition, trimming could be used in combination with orthotics in pathological cases to shift the load away from the diseased digits. With foot disorders being one of the most dehabilitating afflictions affecting the health and well-being of captive elephants, our study could help guide future animal husbandry and foot care techniques and thus contribute to improvements in those areas.

## 5. Conclusion

Using custom-written software, we analysed the foot pressure distributions and COP trajectories between African and Asian elephants reared on natural substrates but with different foot care protocols. We have shown that COP trajectories and mean peak pressure patterns do not differ (admittedly with a descriptive rather than statistically robust sample) between species, further supporting their similarities on locomotor behaviour and mechanics. Variations in pressure magnitudes can be due to age, body mass and measurement variations, among other factors, thus it is not possible to conclude from this study the effect of different foot management approaches or substrate habitats on the elephant foot pressure. Our study is also the first to show that foot pressures in elephants reared on natural substrates are concentrated on the lateral part of their foot. We speculated that exposure to hard substrates and captivity in constrained environments may upset the natural mechanics of the foot, increase foot pressures and induce foot disease on the lateral digits, which are more prone to pathogenesis (also see [4]). We anticipate our data and methods might be used as controls to monitor pressure patterns in captive elephants with the scope to detect abnormalities at early stages and improve elephant husbandry.

## Supplementary Material

Supplementary Data 1. Raw experimental gait data for all African elephants.

## Supplementary Material

Supplementary Data 2. Raw experimental foot pressure data for all African elephants.

## Supplementary Material

Supplementary Data 3. Raw experimental foot pressure data for all Asian elephants (excluding subject 1) by Panagiotopoulou et al., 2012.

## Supplementary Material

Supplementary Data 4. Raw experimental COP data for all African elephants.

## Data accessibility

All raw data are reported as the electronic supplementary material.

## References

[1] Weissengruber GE, Egger GF, Hutchinson JR, Groenewald HB, Elsasser L, Famini D, Forstenpointner G 2006 The structure of the cushions in the feet of African elephants (*Loxodonta africana*). J. Anat. 209, 781–792. (doi:10.1111/j.1469-7580.2006.00648.x) 17118065 10.1111/j.1469-7580.2006.00648.x PMC2048995

[2] Hutchinson JR, Delmer C, Miller CE, Hildebrandt T, Pitsillides AA, Boyde AJ 2011 From flat foot to fat foot: the structure, ontogeny, function and evolution of elephant ‘sixth toes’. Science 344, 1699–1703. (doi:10.1126/science.1211437) 10.1126/science.1211437 22194576

[3] Benz A, Zenker W, Hildebrandt TB, Weissengruber G, Geyer H 2009 About the macroscopic and microscopic morphology of elephants’ hooves (Elephantidae). Verh Ber Erkrg Zootiere 42, 167–170.

[4] Panagiotopoulou O, Pataky TC, Hill Z, Hutchinson JR 2012 Statistical parametric mapping of the regional distribution and ontogenetic scaling of foot pressures during walking in Asian elephants (*Elephas maximus*). J. Exp. Biol. 215, 1584–1593. (doi:10.1242/jeb.065862) 22496296 10.1242/jeb.065862

[5] Rome K 1998 Mechanical properties of the heel pad: current theory and review of the literature. Foot 8, 179–185. (doi:10.1016/S0958-2592(98)90026-8)

[6] Moreno K, Carrano MT, Snyder R 2007 Morphological changes in pedal phalanges through ornithopod dinosaur evolution: a biomechanical approach. J. Morph. 268, 50e63. (doi:10.1002/jmor.10498) 10.1002/jmor.10498 17146773

[7] Fowler ME 2001 An overview of foot conditions in Asian and African elephants. In The elephant's foot (eds Csuti B, Sargent EL, Bechert US), pp. 3–7. Ames, IA: Iowa State University Press.

[8] Fowler ME 2001 Elephant foot care: concluding remarks. In The elephant's foot (eds Csuti B, Sargent EL, Bechert US), pp. 147–149. Ames, IA: Iowa State University Press.

[9] Mikota SK, Sargent EL, Ranglack GS 1994 Medical management of elephants, pp. 147–148. West Bloomfield, MI: Indira Publishing House.

[10] Mikota SK 1999 Diseases of the elephant: a review. In *Erkrankungen der Zootiere: Verhandlungsbericht des 39. Internationalen Symposiums uber die Erkrankungen der Zoo und Wildtiere*, pp. 1–15.

[11] Csuti B, Sargent EL, Bechert US 2001 The elephant's foot: prevention and care of foot conditions in captive Asian and African elephants. Ames, IA: Iowa State University Press.

[12] Fowler ME, Mikota SK 2006 Biology, medicine, and surgery of elephants. Ames, IA: Blackwell Publishing Ltd.

[13] Harris M, Sherwin C, Harris S 2008 The welfare, housing and husbandry of elephants in UK zoos. Final Report, University of Bristol. In *DEFRA Science and Research Project WC05007*. London, UK: Department of Food, the Environment and Rural Affairs.

[14] Miller MA, Hogan JN, Meehan CL 2016 Housing and demographic risk factors impacting foot and musculoskeletal health in African elephants (*Loxodonta africana*) and Asian elephants (*Elephas maximus*) in North American Zoo. PLoS ONE 11, e0155223 (doi:10.1371/journal.pone.0155223) 27415763 10.1371/journal.pone.0155223 PMC4944946

[15] Barker ZE, Leach KA, Whay HR, Bell NJ, Main DCJ 2010 Assessment of lameness prevalence and associated risk factors in dairy herds in England and Wales. J. Dairy Sci. 93, 932–941. (doi:10.3168/jds.2009-2309) 20172213 10.3168/jds.2009-2309

[16] Franck A, de Belie N 2006 Concrete floor-bovine claw contact pressures related to floor roughness and deformation of the claw. J. Dairy Sci. 89, 2952–2964. (doi:10.3168/jds.S0022-0302(06)72567-x) 16840610 10.3168/jds.S0022-0302(06)72567-X

[17] von Houwald FF 2001 Foot problems in Indian rhinoceroses (Rhinoceros unicornis) in zoological gardens: macroscopic and microscopic anatomy, pathology, and evaluation of the causes. EEP Res. Committee Newsletter 8, 54–56.

[18] Buckley C 2001 Captive elephant foot care: natural-habitat husbancdry techniques. In The elephant's foot (eds Csuti B, Sargent EL, Bechert US), pp. 53–55. Ames, IA: Iowa State University Press.

[19] Roocroft A, Oosterhuis J 2001 Foot care for captive elephants. In The elephant's foot (eds Csuti B, Sargent EL, Bechert US), pp. 21–52. Ames, IA: Iowa State University Press.

[20] Fowler ME 2006 Foot Disorders. In Biology, medicine, and surgery of elephants (eds Fowler ME, Mikota SK), pp. 271–290. Ames, IA: Blackwell Publishing Ltd.

[21] Hutchinson JR, Schwerda D, Famini D, Dale RHI, Fischer M, Kram R 2006 The locomotor kinematics of African and Asian elephants: changes with speed and size. J. Exp. Biol. 209, 3812–3827. (doi:10.1242/jeb.02443) 16985198 10.1242/jeb.02443

[22] Ren L, Butler M, Miller C, Schwerda D, Fischer M, Hutchinson JR 2008 The movements of limb segments and joints during locomotion in African and Asian elephants. J. Exp. Biol. 211, 2735–2751. (doi:10.1242/jeb.018820) 18723530 10.1242/jeb.018820

[23] Miller CE, Basu C, Fritsch G, Hildebrandt T, Hutchinson JR 2008 Ontogenetic scaling of foot musculoskeletal anatomy in elephants. J. R. Soc. Interface 5, 465–475. (doi:10.1098/rsif.2007.1220) 17974531 10.1098/rsif.2007.1220 PMC2607390

[24] Pataky TC, Caravaggi P, Savage R, Parker D, Goulermas JY, Sellers WI, Crompton RH 2008 New insights into the plantar pressure correlates of walking speed using edobarographic statistical parametric mapping (pSPM). J. Biomech. 41, 1987–1994. (doi:10.1016/j.jbiomech.2008.03.034) 18501364 10.1016/j.jbiomech.2008.03.034

[25] Kuznetsova A, Brockhoff PB, Christensen RHB 2016 lmertest: tests in linear mixed effects models. R package version 2.0-30. See http://CRAN.R-project.org/package=lmerTest.

[26] Dryden IL 2015 Shapes: statistical shape analysis. R package version 1.1-11.

[27] Alexander RMN, Jayes AS 1983 A dynamic similarity hypothesis for the gaits of quadrupedal mammals. J. Zool. 201, 135–152. (doi:10.1111/j.1469-7998.1983.tb04266.x)

[28] Alexander RMN, Jayes AS, Maloiy GMO, Wathuta EM 1979 Allometry of the limb bones of mammals from shrews (*Sorex*) to elephant (*Loxodonta*). J. Zool. 189, 305–314. (doi:10.1111/j.1469-7998.1979.tb03964.x)

[29] Ren L, Miller CE, Lair R, Hutchinson JR 2010 Integration of biomechanical compliance, leverage, and power in elephant limbs. Proc. Natl Acad. Sci. USA 107, 7078–7082. (doi:10.1073/pnas.0911396107) 20351297 10.1073/pnas.0911396107 PMC2872429

[30] Weissengruber GE, Forstenpointner G 2004 Musculature of the crus and pes of the African elephant (*Loxodonta africana*): insight into semiplantigrade limb architecture. Anat. Embryol. 208, 451–461. (doi:10.1007/s00429-004-0406-1) 15340844 10.1007/s00429-004-0406-1

[31] Weissengruber GE, Forstenpointner G 2004 Shock absorbers and more: design principles of the lower hindlimb in African elephants (*Loxodonta africana*). J Morph. 260, 339.

[32] Lees J, Gardiner J, Usherwood J, Nudds R 2016 Locomotor preferences in terrestrial vertebrates: an online crowdsourcing approach to data collection. Sci. Rep. 6, 28825 (doi:10.1038/srep28825) 27381514 10.1038/srep28825 PMC4933880

[33] Ren L, Hutchinson JR 2008 The three-dimensional locomotor dynamics of African (*Loxodonta africana*) and Asian (*Elephas maximus*) elephants reveal a smooth gait transition at moderate speed. J. R. Soc. Interface 5, 195–211. (doi:10.1098/rsif.2007.1095) 17594960 10.1098/rsif.2007.1095 PMC2705974

[34] Ramsay EC, Henry RW 2001 Anatomy of the elephant foot. In The elephant's foot (eds Csuti B, Sargent EL, Bechert US), pp. 9–12. Ames, IA: Iowa State University Press.

